# Omega-3 fatty acid diglyceride emulsions as a novel injectable acute therapeutic in neonatal hypoxic-ischemic brain injury

**DOI:** 10.1016/j.biopha.2024.116749

**Published:** 2024-06

**Authors:** Hylde Zirpoli, Maria Eugenia Bernis, Hemmen Sabir, Denny Joseph Manual Kollareth, James A. Hamilton, Nasi Huang, Jesse Ng, Sergey A. Sosunov, Ben Gaebler, Vadim S. Ten, Richard J. Deckelbaum

**Affiliations:** a Institute of Human Nutrition, Columbia University Irving Medical Center, New York, NY 10032, USA; b Department of Pediatrics, Vagelos College of Physicians and Surgeons, Columbia University Irving Medical Center, New York, NY 10032, USA; c Department of Neonatology and Pediatric Intensive Care, Children’s Hospital, University of Bonn, Germany; d Deutsches Zentrum für Neurodegenerative Erkrankungen (DZNE), Bonn 53127, Germany; e Department of Physiology & Biophysics, Department of Biomedical Engineering, Boston University School of Medicine, Boston, MA 02215, USA; f Division of Neonatology, Department of Pediatrics, Robert Wood Johnson Medical School, Rutgers University, New Brunswick, NJ 08901, USA; g Perimondo LLC, Florida, NY 10921, USA

**Keywords:** hypoxic-ischemic encephalopathy, omega-3 fatty acids, diglycerides, lipid emulsion, neuroprotection, gliosis

## Abstract

Hypoxic-ischemic encephalopathy (HIE), resulting from a lack of blood flow and oxygen before or during newborn delivery, is a leading cause of cerebral palsy and neurological disability in children. Therapeutic hypothermia (TH), the current standard of care in HIE, is only beneficial in 1 of 7–8 cases. Therefore, there is a critical need for more efficient treatments. We have previously reported that omega-3 (n-3) fatty acids (FA) carried by triglyceride (TG) lipid emulsions provide neuroprotection after experimental hypoxic-ischemic (HI) injury in neonatal mice. Herein, we propose a novel acute therapeutic approach using an n-3 diglyceride (DG) lipid emulsions. Importantly, n-3 DG preparations had much smaller particle size compared to commercially available or lab-made n-3 TG emulsions. We showed that n-3 DG molecules have the advantage of incorporating at substantially higher levels than n-3 TG into an *in vitro* model of phospholipid membranes. We also observed that n-3 DG after parenteral administration in neonatal mice reaches the bloodstream more rapidly than n-3 TG. Using neonatal HI brain injury models in mice and rats, we found that n-3 DG emulsions provide superior neuroprotection than n-3 TG emulsions or TH in decreasing brain infarct size. Additionally, we found that n-3 DGs attenuate microgliosis and astrogliosis. Thus, n-3 DG emulsions are a superior, promising, and novel therapy for treating HIE.

## Highlights

• Therapeutic hypothermia (TH), the standard of care in HIE, has substantial limitations in the clinical practice.
• To improve n-3 FA bioavailability, we formulated a diglyceride (DG) lipid emulsion for acute parenteral administration.
• n-3 DG emulsion showed superior neuroprotection compared to TH and other n-3 based lipid emulsions.

## Graphical Abstract

**Figure:**
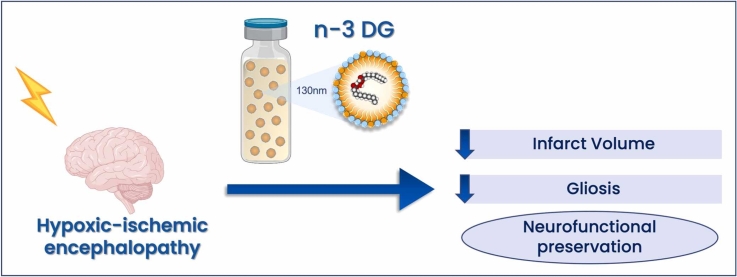

## 1 Introduction

Neonatal hypoxic-ischemic encephalopathy (HIE), caused by a lack of blood flow and oxygen to the brain during labor or delivery, has a global incidence of 1–5 per 1000 live births [1], [2], and is a major cause of mortality, cerebral palsy, and other neurological complications [3], [4], [5]. The accepted standard of care for HIE is therapeutic hypothermia (TH), which involves cooling of the newborn and offers only mildly improved outcomes to 1 in 7–8 affected newborns in the US and Europe, while in lower middle-income countries it can lead to worse outcomes [6], [7], [8], [9], [10], [11], [12], [13]. TH in clinical practice has many challenges and limitations, including variability in clinical response; incomplete protection with many infants still developing cerebral palsy, cognitive impairments, or other neurodevelopmental disabilities; and adverse effects such as electrolyte disturbances, sinus bradycardia, thrombocytopenia, and respiratory complications [14], [15], [16], [17]. Thus, there is a critical need for more accessible and effective HIE treatment globally.

Substantial efforts have been devoted finding an alternative to TH and discovering novel neuroprotective therapies for HIE. Indeed, several experimental studies in animal models as well as clinical trials have provided evidence for oral omega-3 (n-3) fatty acid (FA) therapeutics (Vascepa®, Lovaza®) and dietary supplements as potential cardio- and neuroprotective strategies in cardiovascular diseases [18], [19]. However, oral n-3 FA therapeutics/supplements require weeks-months to modify FA membrane compositions, a clear disadvantage in acute injuries. Herein we focus on acute bolus injections of n-3 FA, which induce rapid increases (minutes-hours) in n-3 FA membrane composition [18], [20], [21], [22], and could offer a new therapeutic approach for using n-3 FAs to treat acute organ injuries.

We have recently reported that acute injection of docosahexaenoic acid (DHA), but not eicosapentaenoic acid (EPA), administered in the form of triglyceride (TG) emulsion particles after HI injury in neonatal mice significantly attenuated brain damage [25]. Our group and others have also characterized pathways regulated by acutely injected n-3 FAs or their Specialized Pro-resolving Mediator derivatives, demonstrating their bioactive and pleiotropic actions. These include (i) mitochondrial reactive oxygen species (ROS) reduction [23], [24], (ii) mitochondrial Ca^2+^ homeostasis preservation [25], (iii) cell death pathways modulation [26], [27], [28], [29], and (iv) inflammatory response attenuation [30], [31], [32]. Furthermore, higher levels of n-3 FAs increase cellular membranes fluidity, facilitating active interactions of receptors, ion channels, and protein complexes [33], [34], [35], [36]. Working separately or synergistically, these mechanisms can contribute to n-3 FA effectiveness in ischemic injuries, lessening inflammation while accelerating repair processes.

The current study investigates the efficacy of a novel parenteral formulation, a diglyceride (DG) lipid emulsion carrying both DHA and EPA. We optimized the preparation of this novel n-3 DG emulsion, analyzed its physical-biochemical properties, and investigated the neuroprotection of n-3 DG compared to n-3 TG emulsions and TH. We also studied the effects of n-3 DG emulsions on reactive gliosis in the subacute phases of HI insult.

As a strength of our studies, two different laboratories at the University of Bonn and Columbia University collaborated to compare the effects of n-3 DG and TG injected emulsions after HI in neonatal rats and mice, respectively, using equivalent assessments for neuroprotective outcomes. The importance of these collaborative results is supported by The Stroke Therapy Academic/Industry Roundtable (STAIR) recommendations [37], in which part of the guidelines include demonstrating efficacy of neuroprotective agents in two or more laboratories in separate institutions and in different animal species. Thus, our physical, biological and efficacy findings support the use of DG emulsions over n-3 TG as a new and more effective carrier for n-3 FAs and as a superior acute intervention for HIE treatment compared to the current standard of care, TH.

## 2 Material and methods

### 2.1 Materials

Tri-DHA (cat # T-310) was purchased from Nu-Chek Prep, Inc. (Elysian, MN). Egg yolk phosphatidylcholine (cat # 840051 C) was obtained from Avanti Polar-Lipids, Inc. (Alabaster, AL). Omegaven® (a triglyceride lipid emulsion containing fish oil) was obtained from Fresenius Kabi AG © (Germany). To produce n-3 DG lipid emulsions, n-3 DG oils were synthesized by DSM (Dartmouth, Nova Scotia), using reverse reactions of lipase-catalyzed glycerolysis (i.e., transesterification) with n-3 long chain FAs. NEFA C, glycerol blanking method and choline oxidase-DAOS method for free fatty acid, glyceride and phospholipid assays respectively were purchased from Wako Chemicals USA, Inc., Richmond, VA. Bovine lipoprotein lipase (LpL) from *Burkholderia sp* was purchased from Millipore-Sigma Inc, USA. BHT (2,6-Di-tert-butyl-4-methoxyphenol), triphenyl-tetrazolium chloride (TTC) and Hematoxylin and eosin (H&E) were obtained from Sigma-Aldrich, St. Louis, MO. Ionized calcium-binding adapter molecule 1 (IBA-1; cat # 019–19741) was purchased from Wako Chemicals USA, Inc., Richmond, VA; glial fibrillary acidic protein (GFAP; cat # 80788) antibodies was purchased from Cell Signaling, Danvers, MA, USA.

### 2.2 Preparation of lipid emulsions

Omegaven® is a triglyceride-based emulsion commercially available, containing 10% TG (10 g TG/100 mL), with EPA content between 1.25 and 2.82 g/100 mL and DHA content between 1.44 and 3.09 g/100 mL, and FDA approved for IV nutrition in pediatric patients with parenteral nutrition-associated cholestasis. For laboratory-made emulsions, we used TG oils containing only DHA, as TG oil with only EPA did not have protective effects [25]. We previously reported that both Omegaven and lab-made n-3 TG have equal neuroprotective effects [24], [25]. The n-3 DG oil contained > 90% of total FA as EPA and DHA, with an EPA/DHA weight ratio of 1.35.

Aiming to prepare 10% (10 g TG or DG /100 mL) lab-made lipid emulsions, comparable to the commercially available Omegaven®, n-3 DG or TG-DHA (referred as n-3 TG) oils, were mixed with solubilized egg yolk phosphatidylcholine (PC) with methods previously described by our Columbia University laboratory [24]. Briefly, H_2_O containing 0.25 mM EDTA and glycerin were mixed at 60°C. Then, PC, sodium oleate and DG or TG oils were added and mixed using a homogenizer at 60°C as a pre-emulsion step. The pre-emulsion was processed either through a high pressure microfluidizer LV1 for five passes at 900-bar pressure, or by sonication for 1 h at 50°C, 140 W under a stream of N_2_ using a Branson Sonifier model 450 (Branson Scientific, Melville, NY). Adjustments to pH 8.5 ± 0.2 were done using 1 N acid or base (HCL or NaOH). We optimized and standardized this procedure, which resulted in highly predictive and reproducible n-3 DG emulsions with mean particle sizes below 130 nm and with DG concentrations of 10 g/100 mL of emulsion. The final emulsion concentrations were analyzed for the amounts of glycerol and PC by enzymatic procedure using GPO-HMMPS, glycerol blanking method and choline oxidase-DAOS method (see above). The emulsions were then stored under argon at 4°C and were used for experiments within 4 weeks of preparation.

### 2.3 Particle size, polydispersity, zeta potential, oxidative measurements, and emulsion stability

Mean particle size (Z-average), zeta potential and polydispersity index (PDI) of DG and TG emulsions were determined via a Malvern Zetasizer ZS90 at a wavelength of 635 nm, with fixed light incidence angle 900 and at 25°C. Before analysis, 10 μL of lipid emulsion was dispersed in 1 mL of de-ionized water, and the sample was introduced into a cuvette for analysis. The measurements were analyzed using Zetasizer family software update v8.02 (Malvern Instruments, Worcestershire, UK).

To assess oxidative stability parameters of the lipid emulsion samples, p-anisidine (p-AV) assays were measured. Prior to analysis, the oil samples were extracted from the emulsions by adding 3 mL of 50 mg/L BHT (2,6-Di-tert-butyl-4-methoxyphenol) in hexane and 3 mL of 3% sodium chloride. The mixture was kept on a stir plate for 30 min, followed by centrifugation at 1100 rpm for 15 min, and the top hexane layer was transferred into a 10 mL scintillation vial to evaporate to dryness under nitrogen. p-AV of the oils and emulsions were determined according to the standard of AOCS Official Method Cd 18–90, using a Thermo Scientific™ GENESYS™ spectrophotometer at 350 nm. Eight weeks and up to six months after the preparation, we further analyzed the stability of DG or TG emulsions by repeating mean particle size, p-AV, PDI measurements, and thin layer chromatography (TLC) to analyze lipid composition. We performed an accelerated stress stability study where we stored the emulsions at 25 °C for six months. Lipids were extracted by hexane/2-propanol and different lipid classes were separated by TLC with the solvent system hexane/diethyl ether/acetic acid (70/30/1 v/v/v). Samples were run in parallel lanes with standards and individual spots were identified by iodine vapor.

### 2.4 *In vitro* studies

#### 2.4.1 NMR – analyses of n-3 DG vs n-3TG in model membranes

##### 2.4.1.1 Sample preparation

Egg phosphatidylcholine (PC) was obtained from Avanti Polar Lipids (Birmingham, AL) and used without further purification. For these analyses, n-3 TG (TG-EPA and TG-DHA) and n-3 DG (DG-DHA) oils were obtained from Nu Chek Prep (Elysian, MN). Small unilamellar vesicles (SUV) were made by sonication of egg yolk PC with increasing amounts of DG-DHA, TG-DHA or TG-EPA, given as mol% of the total lipid. The incorporation of the glyceride components to the PC bilayer was monitored by NMR spectroscopy. Vesicles were prepared as described previously [38], [39] in 0.56% KCl or 0.05 M Tris buffer, pH 7.4, and the sample temperature (measured directly by a thin thermocouple) was kept at 10–20°C during sonication. The amount added for DG or TG ranged between 3 and 40 mol%. All NMR spectra were obtained in 30 min under identical conditions. The NMR spectra recorded the changes in the PC structural organization and molecular motions starting from baseline measurements before addition of DGs or TGs.

##### 2.4.1.2 NMR spectroscopy

Fourier transform NMR spectra were obtained at 50.3 MHz with a Bruker WP200 spectrometer equipped with an Aspect 2000A computer as previously described [40], [41], [42]. NMR spectra of vesicles were obtained initially at 15±5°C before raising the temperature. Spectra of neat DG or TG with H_2_O were obtained after adding a small drop of water with mixing to liquid-phase DG/TG. Spin lattice relaxation times were measured using a fast inversion recovery method and calculated with a three-parameter exponential fitting. Chemical shift and linewidth values were measured digitally. High resolution broad-band decoupled NMR spectra of DG or TG/PC sonicated vesicle samples were obtained with a spectral width of 30,000 Hz, 8192 data points, a 90 pulse, and a 3.0-s pulse interval.

### 2.5 Lipolysis of lipid emulsions

Lipid emulsions (n-3 DG vs n-3 TG) were incubated at 37°C with a buffer (1 M Tris-base, pH 8.6, 20% bovine albumin, 1 mmol/L EDTA, and 100 IU/mL heparin) in the presence or absence of bovine lipoprotein lipase (LpL) from *Burkholderia sp* [43]. The activity of purified LpL was 300–400 U/mg of LpL protein (1 U=1 μmol of free FA (FFA) released from DG or TG/min at 25°C). Purified LpL was diluted 1:100 in 0.9% NaCl at pH 8.6, immediately prior to incubation with emulsions. Human plasma containing apoCII, a cofactor required for activating LpL activity, was added to the incubation mixture as described elsewhere [43]. Experiments were performed with increasing amounts of LpL (0–20 μL of 1:100 dilution) over a fixed time (30 min). Released FFA from the lipid emulsions were measured using an enzymatic kit (NEFA C Kit). Studies were performed in triplicate for each experiment with at least four separate dose-response experiments.

### 2.6 *In vivo* studies

#### 2.6.1 Pharmacokinetics - plasma glyceride levels

All studies in mice were conducted following protocols approved by the Columbia University Institutional Animal Care and Use Committee (IACUC) and in accordance with the Association for Assessment and Accreditation of Laboratory Animal Care (AAALAC) guidelines. Eight-day-old C57BL/6 J neonatal mice with their dams were purchased from Jackson Laboratories (Bar Harbor, ME, USA). We performed pharmacokinetics studies in ten-day-old (P10) mice, by analyzing plasma increases in glyceride levels after either DG or TG lipid emulsion intraperitoneal (IP) injections. We use 0.375 g/kg as a “standard” dose for both emulsions (see below). For blood collection, one animal was used for only one-time point, as neonatal mice only allow terminal methods of bleeds to collect samples of adequate volume. Blood was collected either at 0, 1, 2 or 4 h after injection of the lipid emulsions. Total plasma glyceride was enzymatically measured by GPO-HMMPS, glycerol blanking method.

### 2.7 Unilateral cerebral hypoxia-ischemia injury in mice

**Fig. 1:**
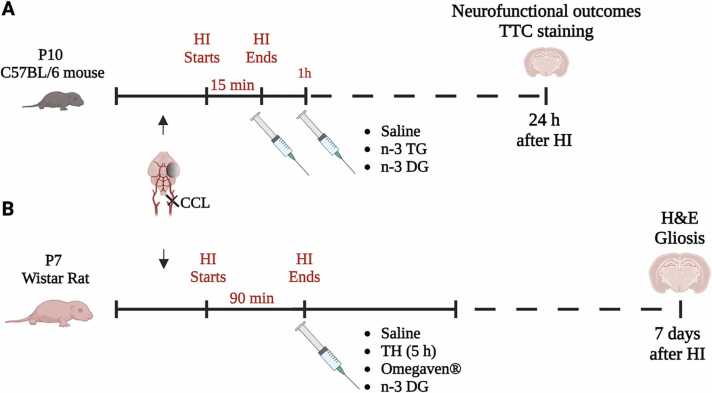
Experimental design using the Rice–Vannucci model for hypoxic-ischemic encephalopathy (HIE) in mice and rats. (A) Ten-day-old (P10) C57BL/6 mice or (B) seven-day-old (P7) Wistar rat pups underwent surgery for ligation of the carotid artery (CCL), allowed to recover for 1.5 h with their dam, then placed in a hypoxia chamber at 8% oxygen for either 15 min (mice) or 120 min (rats), and returned to their dam to recover. *Treatments in mice:* two doses of either saline, n-3 DG or n-3 TG, 1 h apart, were IP injected immediately after HI. *Sample collection in mice:* at 24 h after hypoxic-ischemic (HI) injury, neurofunctional outcomes were performed. Immediately after, animals were euthanized, and brains were collected for TTC staining. *Treatments in rats:* single dose of either saline, Omegaven® or n-3 DG were IP injected immediately after HI. One group received TH for 5 h immediately after HI. *Sample collection in rats:* at 7 days after HI injury, brains were collected for H&E staining and immunofluorescence analyses. Abbreviations: diglyceride, DG; triglyceride, TG; therapeutic hypothermia, TH.

Experimental design and timelines are summarized in Fig. 1A. Three-day-old C57BL/6 J neonatal mice with their dams were purchased from Jackson Laboratories (Bar Harbor, ME, USA). For these experiments, we used a total of ∼8 litters.

At ten days of age (P10), mice were subjected to HI insult following the Vannuci model as previously described [24], [25]. Briefly, HI brain injury was induced by permanent ligation of the right common carotid artery under 2% isoflurane anesthesia. After 1.5 h of recovery, mice were exposed to hypoxic (humidified 8% O2/ 92% N2, Tech Air Inc., NY, USA) insult for 15 min, at 37± 0.3°C. To minimize temperature-related variability in the extent of the brain damage, during the initial 15 h of reperfusion after the insult, mice were kept in an isolette at an ambient temperature of 32°C. No exclusion criteria were applied in this hypoxic-ischemic model. To minimize factors that might be associated with experimental variability of the Vannucci model, we randomized each litter either to control or treated groups. We did not exclude infarct sizes equal to zero for any of the groups. DG or TG groups received two IP doses [24], [25], 0.375 g/kg each, one immediately after HI insult and the second one 1 h later. In data not shown, we have demonstrated that the doses selected in this paper use equimolar amounts of n-3 FAs for both n-3 TG and n-3 DG emulsions, providing maximal neuroprotective effects. Normal saline was used as a vehicle (control group).

### 2.8 Unilateral cerebral hypoxia-ischemia injury in rats

Experimental design and timelines are shown in Fig. 1B. All animal experiments were performed as previously described [44], [45], [46] and in accordance with the animal protection committee of the North Rhine-Westphalia, Germany State Environment Agency (LANUV), following the ARRIVE (Animal Research: Reporting of In Vivo Experiments) guidelines.

For all experiments, seven-day-old Wistar Wistar male and female rat pups were used. Animals were housed at the central animal laboratory of the Deutsche Zentrum für Neurodegenerative Erkrankungen (DZNE) in Bonn, Germany, with a 12/12 h dark/light cycle at an environmental temperature of 21°C and with food and water ad libitum. In these experiments, we used a total of ∼12 litters. Prior to the experiments, animals were randomized by litter, sex, and weight to one of the different treatment groups: Saline, TH, Omegaven® and n-3 DG. Two animals were used as sentinels carrying temperature probes: n = 1 for normothermia and n = 1 for TH. These animals were not included in later analyses. Briefly, after subcutaneous injection with 0.05 mg/kg bodyweight (BW) buprenorphine, animals from the HI group also followed Vannucci model protocols and underwent ligation of the left common carotid artery under general anesthesia induced by 5% isoflurane. The HI group was then transferred to a temperature-controlled chamber and exposed to hypoxia at 8% O_2_ for 90 min at a rectal temperature of 36°C. Sentinel pups carried a rectal probe (IT-21, Physitemp Instruments, Clifton, NJ, USA) connected to a servo-controlled cooling machine (CritiCool, MTRE, Yavne, Israel), which controlled a water-filled mat at the base of the chamber. Hypoxia was followed by 5 h of normothermia (NT) (rectal temperature = 37°C) or TH (rectal temperature = 32°C). Normal saline was used as a vehicle (control group). Initial experiments performed were performed comparing 1 dose vs 2 doses. As the results showed the same potency, we proceeded with experiments using only one IP injection. n-3 DG or Omegaven® treated animals received a single dose (0.4 g/kg) administered intraperitoneally immediately after HI insult. Our mortality rate was <15% (in both mice and rats), within the standards for this model, as a result of hypoxia exposure and not due to the treatment.

### 2.9 Neurofunctional outcomes in mice

All neurofunctional outcomes were compared among the study groups and performed by an investigator blinded to the experimental groups. To test coordination and motor ability, righting reflex and negative geotaxis reflex performances were assessed at 24 h after HI injury, as previously detailed [25], [28]. These tests are recommended in rodents before fourteen-day-old, as they are primary reflexes which disappear with age [47]. We included age-matched naïve mice that did not undergo any surgical manipulation and were from the same litters of the treated HI groups, as an additional control group for these analyses.

### 2.10 Measurement of cerebral infarct size in mice and rats

After 24 h reperfusion, mouse brains were collected immediately after performing behavioral tests, coronally sectioned (1 mm thickness) and stained with 2% triphenyl-tetrazolium chloride (TTC) [25], [28]. Unstained areas (negative TTC stains) that appeared white were defined as infarct regions whereas viable regions appeared red. Infarct sizes were calculated using digital images (Adobe Photoshop 4.0.1 and NIH Image J 1.62) and expressed as the percentage of area loss = (1−Area ipsilateral/Area contralateral) × 100.

After sacrifice and transcardial perfusion at P14 (seven days after reperfusion), rat brains were post-fixed in 4% paraformaldehyde overnight at 4°C. The samples were embedded in paraffin and cut into 10 µm coronal sections (Bregma, −0.3 mm and −3.8 mm). Hematoxylin and eosin (H&E) staining was performed for analysis of brain area loss. H&E staining was performed following previously reported standard protocols [44], [48]. The sizes of the ipsilateral (left) and the contralateral (right) hemisphere were measured using NIH Image J 1.62 and area loss calculated as above.

Our laboratories adopted two methods for infarct quantifications, TTC and H&E staining. These are strongly correlated, as previously defined in well-constructed comparative studies [49], [50], [51], [52]. Both TTC and H&E interrogate the tissue morphological alterations and identify dead or dying cells. Each technique is most useful within a specific time frame and complement each other.

### 2.11 Immunofluorescence analysis for microglia and astrocytes markers

Ionized calcium-binding adapter molecule 1 (IBA-1), a specific marker used for microglia, and glial fibrillary acidic protein (GFAP), a specific marker used for astrocytes, were used in these analyses. After fixation with 4% paraformaldehyde overnight at 4 °C, coronal slices of the brain samples were cut with a thickness of 10 µm at a bregma distance of −3.8 mm (area with the most pronounced damage in the model) [45]. The slices were rehydrated by decreasing alcohol concentrations [45]. Thereafter, antigen retrieval was performed by boiling in PBS for 7 min, followed by permeabilization of the cell membrane with 0.1% Triton X-100 for 30 min at room temperature. Slices were blocked with 20% v/v (vol/vol) normal goat serum (Invitrogen, Darmstadt, Germany). The primary antibodies (anti-IBA-1 and anti-GFAP) were incubated overnight at 4 °C followed by incubation of the corresponding secondary antibody for 1 h at room temperature. The slices were counterstained with 4,6-diamidino-2-phenylindole (DAPI) (Invitrogen, Germany). The slices were analyzed by AxioScan.Z1 (Carl Zeiss Microscopy GmbH, Oberkochen, Germany). ZEN Blue 3.1 (Carl Zeiss Microscopy GmbH, Germany) and NIH Image J 1.62 were used for analysis. A fixed area was drawn in different regions at the same coronal section (Bregma −3.8 mm). The areas of interest were cortex (ctx), hippocampus (hipp), and thalamus (th). Z-stack was performed using an interval of 9 slices with a 1 µm distance between each respective z-stack. Maximal projection was used to analyze and quantify both microglia and astrocytes, where the positive cells for the corresponding antibody were matched with their respective nuclear staining, DAPI, and counted.

### 2.12 Statistical analysis

GraphPad Prism 9.1.2 (GraphPad Software, San Diego, CA, USA) was used to analyze and plot the data. Mann–Whitney *U* test was performed when the distribution of the data was non-normal. All data are represented as means ±SEM. One-way ANOVA, two-way ANOVA or Student’s t-test (where appropriate) were used to determine statistically significant differences among naïve, control, TH or the different lipid emulsion groups. Tukey's multiple comparisons test was used to determine p-value.

## 3 Results

### 3.1 n-3 DG emulsions show smaller particle size than n-3TG emulsions

**Fig. 2:**
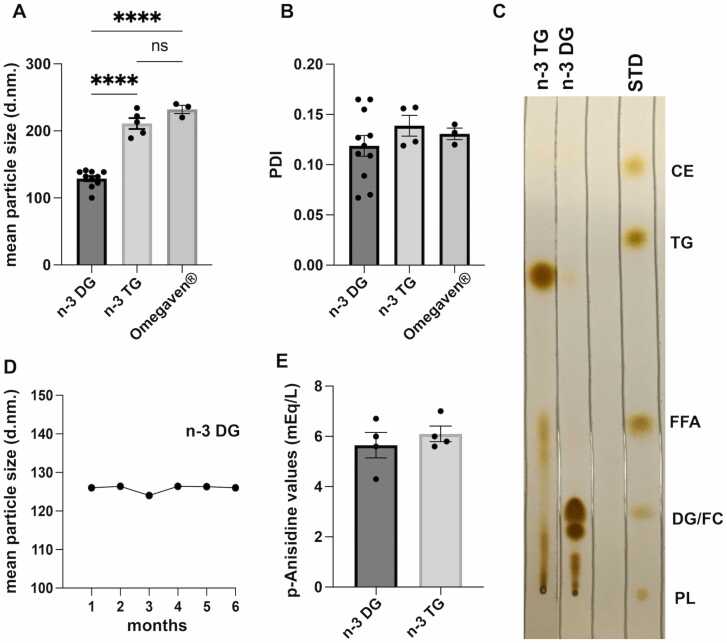
n-3 DG emulsions have smaller particle size and are more stable than n-3TG. (A) Mean particle size (z-average) of diglyceride (DG), triglyceride (TG) and Omegaven®️ emulsions after the preparation procedure; n = 3–10. ****p<0.0001 (one-way ANOVA followed by Tukey's multiple comparisons test); ns: nonsignificant. (B) polydispersity index (PDI) values of DG, TG and Omegaven®️ emulsions; n = 3–10. (C) TLC assay of n-3 DG and TG emulsions; STD = standard: CE, cholesterol ester; TG; FFA, free fatty acids; DG; PL, phospholipids. (D) Mean particle size (z-average) values of DG emulsions stored over 6-month period at 25 °C after an accelerated stress stability study; n = 3–10. (E) p-anisidine values of n-3 DG and TG emulsions; n = 3–5.

We compared lab-made lipid emulsions, prepared using the same method of preparation (see above) with either n-3 DG or n-3TG oils, to commercially available Omegaven® (Fig. 2). The emulsions were characterized by dynamic light scattering (DLS), assessing Z-average diameters (mean particle size), zeta potential and polydispersity index (PDI – homogeneity). n-3 DG preparations had much smaller particle sizes (∼130 nm) compared to the commercially available Omegaven® or lab-made TG, >200 nm (Fig. 2A). In addition, both n-3TG and n-3 DG emulsions were homogenous with similar low PDI values (Fig. 2B). At 4–8 weeks after preparation, thin-layer chromatography (TLC) patterns did not change for DG emulsions (Fig. 2C); while n-3 TG lab-made emulsions showed the presence of FFAs, indicating spontaneous hydrolysis, while this fraction was not detected in DG emulsions (Fig. 2C). We measured particle size in DG emulsion after a 6 months’ accelerated stability study with the emulsion stored at 25°C and we found no detectable deterioration of the emulsion (Fig. 2D), or changes in other parameters. We also analyzed the quality of the oils by measuring p-anisidine values (p-AV) as an index of secondary oxidation compounds. As per the international guidelines [53], all our oils and emulsions had a favorable value of p-AV below 20 mEq/l (Fig. 2E), safe to administer in rodents. We found in an experiment performed in triplicate that the zeta potential for TGs was −35 mV, while for DGs was more negative, −50 mV (data not shown).

### 3.2 *In vitro* studies

#### 3.2.1 n-3 DG molecules incorporate into model membranes more efficiently than n-3TG

**Fig. 3:**
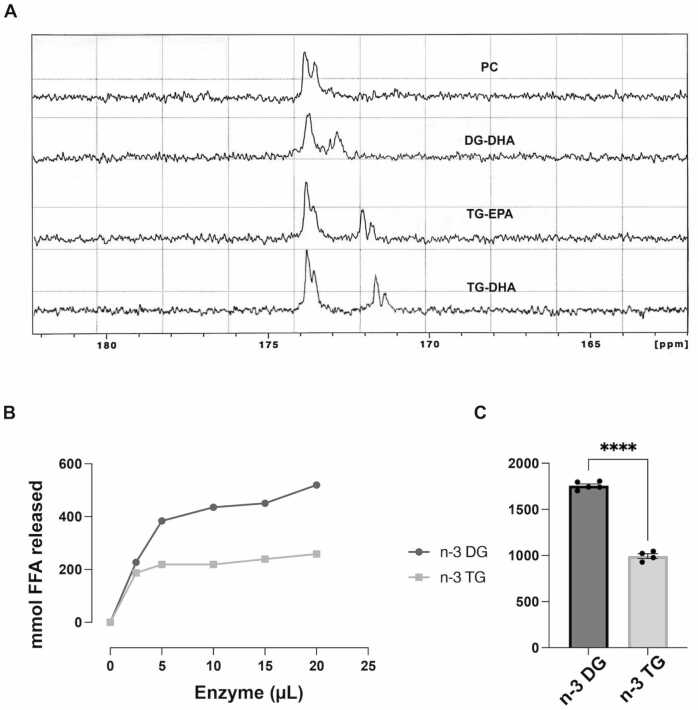
n-3 DGs incorporate in membrane systems more efficiently and are hydrolyzed better than n-3 TG.(A) Representative NMR spectra (carbonyl region) of vesicles with phosphatidylcholine (PC) and 20 mol% DG or TG molecules. Spectra are represented in the following order: PC – DG-DHA – TG-EPA – TG-DHA. (B) and (C) LpL-mediated hydrolysis of n-3 DG vs n-3TG. Experiments were performed with increasing amounts of LpL (0–20 μL of 1:100 dilution) over a fixed time (30 min); data shown are mmol FFA release on the left (B), and FFA areas under the curve on the right (C). n = 4–5. ****p<0.001.

We compared the effects of FAs carried by either DG or TG in model membrane interactions. To evaluate the solubility of DGs and TGs in membrane systems, we studied mixtures of DG (DG-DHA) or TG (TG-DHA and TG-EPA) with phosphatidylcholine (PC) bilayers and analyzed how DGs or TGs incorporate into the lipid mixture and change the properties of the PC structure (Fig. 3A). We did not observe changes in the aliphatic carbons of the PC, which indicates that there was no detectable effect on the phospholipid acyl chains. Therefore, we focused on the polar interface by examination and comparison of the carbonyl region highlighted in each spectrum (Fig. 3A). As shown in a representative experiment, Fig. 3A, the NMR spectrum of SUV with only PC was characterized by relatively narrow peaks indicating rapid molecular motion (high fluidity). The carbonyl region showed two separate peaks, the inner and outer leaflets of the SUV. The higher ppm (downfield) of the outer peak reflected its higher hydration. Compared to TG-DHA, the presence of DG-DHA resulted in a single broader peak indicating that the n-3 DG restricted the mobility of the PC by incorporating into the bilayers. DG-DHA also showed a peak at lower ppm, immediately adjacent to the PC bilayer, suggesting different interfacial interactions with PC. DG peaks did not show different signals in the leaflets of the bilayer, indicating very rapidly flip-flop and higher mobility of the DGs [38], in line with the smaller diameter in DG emulsion particles compared to TG. This markedly differed from n-3 TG, which showed little or no interaction with the SUV system and it separated into an oil phase that was less hydrated and distant from the PC carbonyls, reflecting the lager diameter in emulsion particles we observed for TG (see results below). We could not assess DG-EPA NMR spectra since it is not yet available from Nu-Chek Prep.

#### 3.2.2 LPL-mediated hydrolysis is more efficient in n-3 DG versus TG emulsions

We previously reported very limited lipolysis of n-3 TG compared to n-6 TG emulsions [43]. Here, we assessed LpL-mediated hydrolysis of n-3 DG versus n-3 TG. We predicted that with more DG available at the particle surface than TG, DG emulsions would be better hydrolyzed by LpL. The basal free fatty acid (FFA) levels in these emulsions were similar in the absence of LpL. Experiments were performed with increasing amounts of LpL over a fixed time, 30 min. In these dose-response experiments (Fig. 3B and C), highest lipolysis by LpL occurred in n-3 DG compared to TG emulsions as shown by the area under the curve analysis. There was 1.5-fold more FFA released from n-3 DG compared to n-3 TG emulsions.

### 3.3 *In vivo* studies

#### 3.3.1 n-3 DG emulsions increases the plasma levels of glyceride more rapidly than n-3TG

**Fig. 4:**
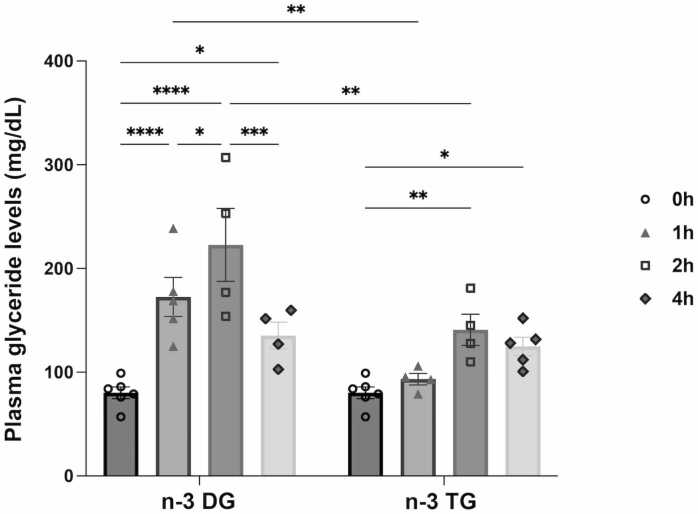
n-3 DG emulsions enter the systemic circulation more rapidly than n-3 TG after IP injection in neonatal mice. P10 non-fasting mice were IP injected with either n-3 DG or n-3TG emulsion (0.375 g/kg). Blood was collected either at 0, 1, 2 or 4 h after injection of the lipid emulsions. Ordinary two-away ANOVA (mixed-effects model) followed by uncorrected Fisher’s LSD was performed (n =4- 5 in each group); *p<0,05, **p<0.01, ***p<0.001, ****p<0.0001. Each bar is the mean ± SEM with the representation of individual values.

To determine whether n-3 DG or TG from the lipid emulsions were systemically absorbed, we examined plasma glyceride levels up to 4 h after IP injection in neonatal mice. After n-3 DG injection, there was a substantial increase of glyceride levels at 1 h time-point, reaching a peak at 2 h with a 3-fold increase (p<0.0001) compared to baseline, followed by a decrease of levels to baseline at 4 h (Fig. 4). This indicates that n-3 DG emulsions had entered the bloodstream and were being catabolized. In comparison, plasma glyceride levels in n-3 TG-injected mice remained similar to baseline at 1 h and showed a peak at later time-points, 2 h (p<0.01) and 4 h (p<0.05), reflecting a slower absorption of n-3 TG compared to n-3 DG.

#### 3.3.2 n-3 DG emulsions decrease infarct size after neonatal HI brain and n-3 DG provide superior neuroprotection compared to n-3TG and hypothermia

**Fig. 5:**
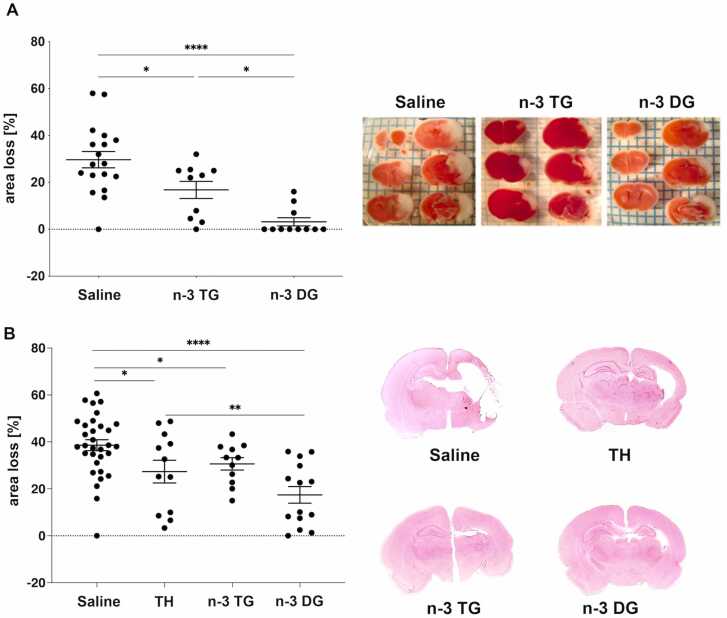
n-3 DG emulsions show stronger neuroprotection than n-3 TG and TH. (A) Individual values and percentage of tissue loss in saline (n=18), n-3 TG (n=10) and n-3 DG (n=11) treated mice after hypoxic-ischemic injury. Values are mean ± SEM. *p<0.05; ****p<0.0001 (ordinary one-way ANOVA followed by Tukey's multiple comparisons test). Representative images of TTC staining for saline, n-3TG, and n-3 DG treated groups. (B) Individual values and percentage of tissue loss in saline (n=32), n-3TG, Omegaven® (n=12), TH (n=11), and n-3 DG (n=14) treated rats after hypoxic-ischemic injury. Values are mean ± SEM. *p<0.05; **p<0.01. Representative images of H&E staining for saline, TH, n-3TG, and n-3 DG treated groups.

We compared the ability of n-3 DG and n-3TG to provide neuroprotection in mice and rats (Fig. 5). Our data showed that neonatal mice treated with n-3 DG exhibited significant reductions in infarct size, by up to 87%, while n-3TG injections decreased the damage by 43%. N-3 DG was at least 3-fold far more effective than n-3TG emulsions (p<0.05) (Fig. 5A), as illustrated by representative TTC staining. In the rat model, the DG emulsion also showed highest efficacy by reducing infarct size by 50% compared to a reduction of 29% after n-3TG treatment and 21% after TH (Fig. 5B), as illustrated by representative H&E staining. Together, these data confirm that n-3 DG has significant neuroprotective effects in both animal models, with n-3 DG emulsions having superior protective properties than n-3 TG or TH.

#### 3.3.3 n-3 DG emulsions preserve short-term neurological outcomes in mice

**Fig. 6:**
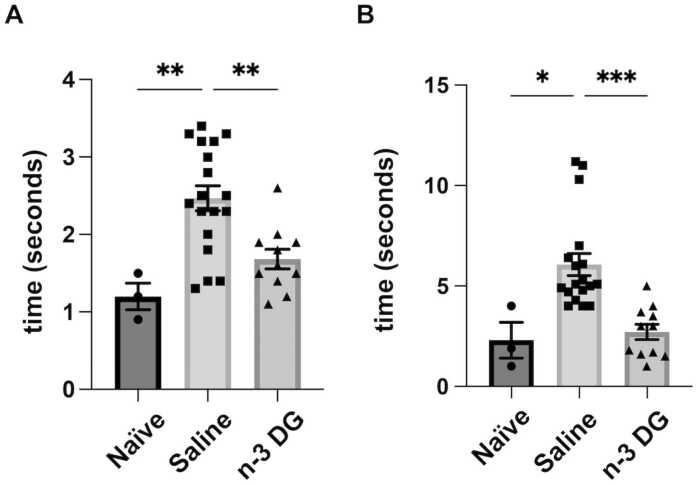
n-3 DG emulsions preserve neurofunctional outcomes in mice. (A) Righting reflex and (B) Negative geotaxis performances in neonatal mice subjected to ischemic injury and acutely treated with n-3 DG emulsions. Each bar represents the mean ± SEM with representation of individual values. Naïve (n=3); Saline (n=18); n-3 DG (n=11). * p<0.05, ** p<0.01; *** p<0.001 (ordinary one-way ANOVA followed by Tukey's multiple comparisons test).

We previously reported that n-3TG emulsions preserve neurofunctional outcomes in neonatal mice after 24 h and up to 8 weeks after HI [25]. We now examined the ability of n-3 DG emulsions to maintain neurofunction in short-term experiments after 24 h reperfusion (Fig. 6). We assessed motor coordination deficits in the control group, showing markedly impaired responses in both negative geotaxis (Fig. 6A), and righting reflexes (Fig. 6B). With DG emulsion treatment, reflex performances were maintained similar to that of naïve mice. Thus, n-3 DG emulsions not only prevent tissue death, but also preserve neurofunctional outcomes.

### 3.4 DG emulsions reduce gliosis after HIE

**Fig. 7:**
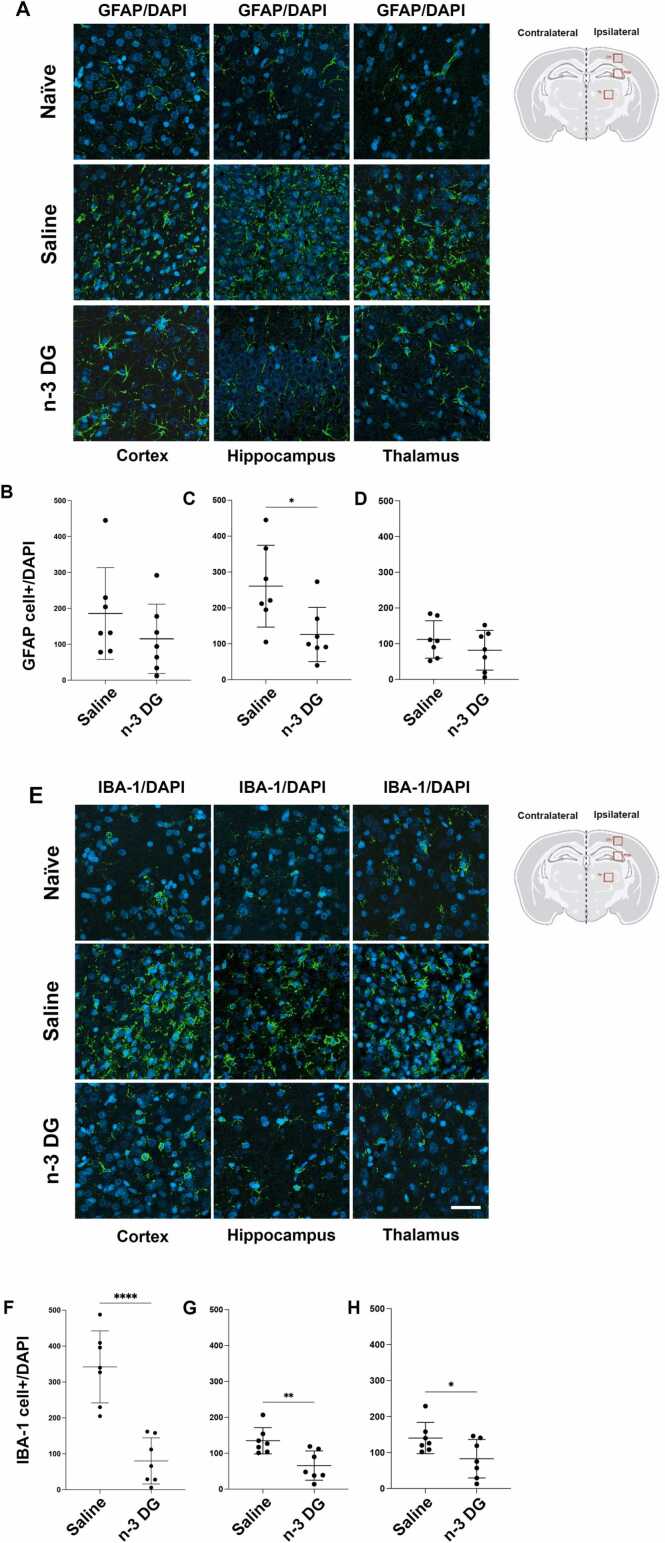
n-3 DG emulsions modulate astrogliosis and microgliosis after HI injury in rats. (A) Representative immunostaining of astrocyte cells, GFAP/DAPI+ cells, for naïve, saline, and n-3 DG treated groups in cortex, hippocampus, and thalamus. (B-D) Quantitative analysis (n=7) of GFAP that co-localized with DAPI+ cells in cortex, hippocampus, and thalamus; scale bar = 20 µm. (E) Representative immunostaining of microglia cells, IBA-1/DAPI+ cells, for naïve, saline, and n-3 DG treated groups in cortex, hippocampus, and thalamus. (B-D) Quantitative analysis (n=7) of IBA-1 that co-localized with DAPI+ cells in cortex, hippocampus, and thalamus; scale bar = 20 µm. Values are means ± SEM. Mann–Whitney U tests was performed, with * p<0.05, ** p<0.01; **** p<0.0001.

In the neonatal HI rat model, using the specific marker for astrocytes, GFAP, we observed that after 7-days post HI, astrogliosis was significantly reduced in the hippocampal area after treatment with n-3 DG (Fig. 7 A and C) compared to the saline group. The cortical area and thalamus showed a slight but not significant decrease in astrogliosis when compared to the saline group (Fig. 7 A, B, and D). Interestingly, n-3 DG treatment significantly attenuated microgliosis, as shown by using the specific microglia marker IBA-1, in the three areas analyzed, when compared to the saline group (Fig. 7E-H). n-3 DG treatment resulted in similar staining patterns for IBA-1 as in naïve brains (Fig. 7E). Thus, injection of n-3 DGs significantly affects reactive gliosis, attenuating further progression of brain lesions after HI injury.

## 4 Discussion

A large body of evidence shows that n-3 FAs have complex structural and functional roles in the central nervous system (CNS), which may be mediated by direct regulation of various cellular targets or through the production of their bioactive metabolites and lipid mediators [18], [23], [54], [55], [56]. We initially provided evidence that n-3 FAs packaged in TG emulsions have significant potential as a rapidly acting acute intervention in ischemic injury in heart and brain [25], [29]. However, our current study offers a novel and better formulation, n-3 FAs carried by a DG lipid emulsion, that has the advantage of reducing brain injury even more than n-3 TG, demonstrated at both neuropathological and neurobehavioral levels. Importantly, we observed that n-3 DG shows marked superiority in reducing infarct areas compared to the current standard of care, TH. We found that n-3 DG attenuated microgliosis and astrogliosis in the subacute phase of the injury. Thus, n-3 DG treatment promptly intervenes in the HI insult cascade to confer neuroprotection and to activate cytoprotective mechanisms in response to the brain injury.

Particle size distribution and stability are key factors for the safety and efficacy of emulsion preparations [57], [58], [59], [60]. Our studies have led to the development of stable n-3 DG emulsions that exhibit comparable or even better characteristics than commercially available n-3TG emulsions. n-3 DG molecules have an inherent advantage over natural fish oil packed in TGs, as they are more polar and likely act as self-emulsifiers, positioning themselves at the aqueous-oil interface. This property can also contribute to our observation that, under identical preparation conditions, we obtained smaller particle size for n-3 DGs than for n-3 TGs. Nanosized <200 nm particle delivery systems have emerged as promising strategies for more efficient and rapid absorption across the blood-brain barrier (BBB) and entry into brain cells [57], [59], [61], [62]. Thus, our n-3 DG emulsions, with particle sizes smaller than 130 nm, represent a potential improved delivery system to enhance the bioavailability of n-3 FAs to the brain, resulting in accelerated molecular actions that modulate neuroprotective pathways.

Furthermore, our NMR results confirm that n-3 DG molecules do not disrupt the lipid bilayer membrane structure in our *in vitro* model and are better incorporated into PC membrane systems than n-3 TG, allowing a faster trans-bilayer membrane movement. We suggest that this phenomenon could potentially facilitate a more rapid modulation on intracellular pathways involved in neuroprotection. The more hydrophilic nature of DGs, compared to TGs, also provides higher incorporation into the emulsion surface where hydrolysis by LpL occurs. Dr. Ginsberg’s group [63] previously found that clearance of DG-chylomicrons, after oral or intravenous DG administration (enriched with oleic and linoleic acids), was more rapid than that of TG-chylomicrons; this was associated with more efficient in vitro LpL-mediated lipolysis of DG-derived chylomicrons. Intravenously infused DG was also cleared faster than TG in mice, via both LpL-mediated lipolysis and apolipoprotein E (apoE)-dependent hepatic uptake, in line with our pharmacokinetics and *in vitro* LpL results. Here, we propose that the faster hydrolysis and bloodstream absorption of n-3 DGs contribute to the difference in neuroprotective effects observed with n-3 TG. Because of n-3 DG capability to affect both structural and mobility dynamics in phospholipid bilayers, combined with their smaller particle size, DG emulsions hold potential as a platform for enhanced delivery of n-3 FAs and derivatives to the brain.

Our PK experiments also demonstrated that n-3 DG emulsions are systemically absorbed after IP injection with faster kinetics than TG emulsions. Still, we acknowledge that this study does not offer direct assessments of n-3 DG crossing BBB; indeed, future studies will assess potential changes in n-3 FAs content in brain after DG versus TG administration.

We previously reported that TH and n-3 TG treatment reduced infarct volume in neonatal mice by 45% compared to the control group exposed to normothermia, using the same settings of ischemic injury reported in this study [64]. In the current study, we confirmed similar neuroprotection using the same n-3 TG treatment (45% reduction), while neonatal mice treated with n-3 DG had a decreased infarct size by up to 87%. Thus, in our models, with somewhat different approaches, both in rats and mice, n-3 DG is far more effective than TH and n-3 TG.

Not all the compounds effective in mice are neuroprotective in rats and vice versa. Dose or route of administration could change among experiments in different species. As an example, using a mouse model of carotid occlusion, Matsuoka et al. showed that intra-venous administration of catechin improved behavioral deficits [65]. In contrast, catechin injection after ischemia did not give protection using a permanent occlusion model in rats [66]. In our study, following some of the STAIR guidelines [37], we demonstrate herein the reproducibility of n-3 DG results in two different laboratories and in two species. Importantly, as indicators of efficient catabolism and safety of the emulsion, after injection in the studies herein (and in ongoing early studies in neonatal piglets) there are no signs of prolonged bleeding and we do not observe any lipid emulsion accumulation in lungs or any respiratory symptoms.

The priming of microglial cells is influenced by a complex interplay of signals from neighboring cells, such as neurons, astrocytes, and endothelial cells. The ischemic injury disrupts the integrity of these cell networks and serves as a trigger for reactive astrogliosis and upregulation of GFAP [67], [68], [69], [70], [71]. Astrocyte hyperactivity is associated with secondary neuronal damage due to the release of pro-inflammatory cytokines after HI. Several studies have shown that n-3 FAs improve antioxidant defense and preserve mitochondrial functionality in astrocytes [72], [73], [74], [75]. Here, our immunostaining data identified a conspicuous astrocytic response after ischemic injury, partially reversed by n-3 DG treatment. This correlates with previous data demonstrating that exposure to n-3 FAs could regulate glial activation particularly in aging and neurodegenerative diseases [54], [76]. Furthermore, the anti-inflammatory effects of n-3 DGs on microglia may have important implications for the treatment of ischemic brain injury and other neuroinflammatory conditions. Indeed, recent evidence has demonstrated that systemic administration of n-3 FAs can significantly reduce neuronal and glial cell death, improving locomotor recovery after acute injuries related to the heart, brain, and spinal cord [18]. Here, we showed that n-3 DG treatment affects inflammation markers in the injured area by reducing both GFAP and IBA-1 levels in the subacute phases of HI injury. As a limitation of our study, we are aware that these markers are indicative of microgliosis and astrogliosis, but do not offer a complete description of the inflammatory pathways involved after ischemic insult, and that the downregulation observed in GFAP and IBA-1 levels does not define the direct effect of n-3 DGs on gliosis. However, the reduction in these markers observed after DG administration, which associates with the neuroprotective effects of n-3 DG, provides initial, but still important evidence that will help direct future studies. We will examine the pro/anti-inflammatory cytokines cascade in time-dependent manners as well as morphological analyses of glial cells, contributing to better understanding of the mechanisms underlying the effects of n-3 DGs in neuroinflammation during the acute and subacute phases of ischemic damage.

## 5 Conclusion

Our findings in two rodent models show that neuronal death and tissue damage elicited by HIE can be ameliorated by acute n-3 DG treatment, along with preservation of neurofunctional outcomes and reduction in reactive gliosis. n-3 DG emulsions (i) have smaller particle size, (ii) incorporate to a higher extent in the interfacial surface of phospholipid membrane systems, and (iii) as we have shown, this facilitates a more rapid hydrolysis of n-3 DG compared to n-3 TG emulsions. These properties make DG a better carrier for n-3 FAs, potentiating their strong neuroprotective effects. n-3 DG use in other acute conditions, such as traumatic brain injury or ischemic stroke, should also be investigated along with long-term neurofunctional benefits by n-3 DG, so that clinical translation can be successful in fields awaiting major therapeutic advances for neuroprotection.

## Informed consent statement

Not applicable.

## Author Contributions

Conceived the project: H.Z. and R.J.D. Designed experiments: H.Z., R.J.D., V.S.T., H.S. Generated data for most of the experiments: H.Z., M.E.B. Conducted/analyzed mouse studies: H.Z., S.A.S., D.J.M.K. Conducted/analyzed rat studies: M.E.B. and H.S. Conduct-ed/analyzed NMR: J.A.H., N.H., J.N. Conducted/analyzed emulsion characterization: H.Z., B.G. Wrote the manuscript: H.Z. and R.J.D. Critically edited the manuscript: H.Z., R.J.D., J.A.H., M.E.B., H.S. Approved final version of the manuscript: all authors.

## CRediT authorship contribution statement

**Jesse Ng:** Methodology. **Sergey A Sosunov:** Methodology. **Ben Gaebler:** Methodology. **Vadim S Ten:** Writing – review & editing, Visualization, Supervision, Formal analysis, Data curation. **Richard J Deckelbaum:** Writing – review & editing, Supervision, Project administration, Investigation, Conceptualization. **Hylde Zirpoli:** Writing – original draft, Supervision, Methodology, Investigation, Formal analysis, Data curation, Conceptualization. **Maria Eugenia Bernis:** Writing – review & editing, Methodology, Formal analysis, Data curation. **Hemmen Sabir:** Writing – review & editing, Visualization, Investigation, Funding acquisition, Formal analysis, Data curation. **Denny Joseph Manual Kollareth:** Methodology. **James A Hamilton:** Writing – review & editing, Methodology, Data curation. **Nasi Huang:** Methodology.

## Declaration of Competing Interest

Richard J Deckelbaum is a founding scientist and scientific advisory board member of DeckTherapeutics Inc., a company that plans to use novel n-3 lipid emulsions to prevent tissue death after ischemic brain injury. Hylde Zirpoli is a scientific advisory board member of DeckTherapeutics Inc. DeckTherapeutics Inc. had no inputs or roles in the experimental design, data analysis and funding of this paper. The other authors declare no conflict of interest.
